# Expression and Function of Thyroid Hormone Transporters in the Microvillous Plasma Membrane of Human Term Placental Syncytiotrophoblast

**DOI:** 10.1210/en.2012-1753

**Published:** 2012-10-18

**Authors:** L. S. Loubière, E. Vasilopoulou, J. D. Glazier, P. M. Taylor, J. A. Franklyn, M. D. Kilby, Shiao Y. Chan

**Affiliations:** School of Clinical and Experimental Medicine (L.S.L., E.V., J.A.F., M.D.K., S.Y.C.), College of Medical and Dental Sciences, University of Birmingham, Birmingham B15 2TT, United Kingdom; Fetal Medicine Centre (M.D.K.), Birmingham Women's Foundation Trust, Edgbaston, Birmingham, B15 2TG, United Kingdom; Maternal and Fetal Health Research Centre (J.D.G.), School of Biomedicine, Manchester Academic Health Science Centre, University of Manchester, St Mary's Hospital, Manchester M13 9WL, United Kingdom; and Division of Cell Signalling and Immunology (P.M.T.), University of Dundee, Dundee DD1 5EH, United Kingdom

## Abstract

The transplacental passage of thyroid hormones (THs) from mother to fetus in humans has been deduced from observational clinical studies and is important for normal fetoplacental development. To investigate the transporters that regulate TH uptake by syncytiotrophoblast (the primary barrier to maternal-fetal exchange, which lies in direct contact with maternal blood), we isolated the microvillous plasma membrane (MVM) of human term syncytiotrophoblasts. We have demonstrated that MVM vesicles express plasma membrane TH transporter proteins, including system-L (L-type amino acid transporter 1 and CD98), monocarboxylate transporters (MCTs) 8 and 10, organic anion-transporting polypeptides 1A2 and 4A1. We provide the first definitive evidence that the human syncytiotrophoblast MVM is capable of rapid, saturable T_4_ and T_3_ uptake at similar rates and in a Na^+^-independent manner. These two major forms of THs could not significantly inhibit each others' uptake, suggesting that each is mediated by largely different transporters. No single transporter was noted to play a dominant role in either T_4_ or T_3_ uptake. Using combinations of transporter inhibitors that had an additive effect on TH uptake, we provide evidence that 67% of saturable T_4_ uptake is facilitated by system-L and MCT10 with a minor role played by organic anion-transporting polypeptides, whereas 87% of saturable T_3_ uptake is mediated by MCT8 and MCT10. Our data demonstrate that syncytiotrophoblast may control the quantity and forms of THs taken up by the human placenta. Thus, syncytiotrophoblast could be critical in regulating transplacental TH supply from the mother to the fetus.

Associations between minor abnormalities of maternal thyroid function in early pregnancy and long-term neurodevelopmental delay in offspring (1–3) have led to the widely held view that the transplacental passage of maternal thyroid hormones (THs) is critical to normal human fetal central nervous system development. These effects are most pertinent when maternal thyroid dysfunction is present before the onset of endogenous fetal TH production in the second trimester of pregnancy (4). The transplacental passage of maternal THs from very early gestation in humans has been deduced from several observational studies, although the mechanisms involved and how it may be regulated remain incompletely understood (5).

Before endogenous fetal TH production, T_4_ has been found in the exocoelomic cavity (fetal compartment) from 5 wk of gestation (6). Both T_3_ and T_4_ have been found in fetal limbs by 6–8 wk and in fetal brain and liver by 9–12 wk of gestation (7). The human fetal cerebral cortex expresses functional TH transporters, TH receptors, and deiodinase enzymes from 7 wk gestation and, thus, has the necessary molecular apparatus to respond to maternal THs from early gestation (8, 9). The transplacental passage of maternal THs could continue until delivery, because athyroidal human fetuses born at term have (maternally derived) circulating T_4_ at concentrations of 25–50% of normal-term fetuses (10). Furthermore, very premature neonates have lower circulating TH concentrations than that of their *in utero* counterparts of the same age, a finding which is postulated to be due to the sudden termination of the transplacental supply of maternal THs and iodide (11).

There is also evidence for an additional impact of maternal thyroid dysfunction directly upon placental development itself. *In vitro* studies have demonstrated that THs influence villous and extravillous trophoblast proliferation, viability, and invasive ability (12, 13). Thus, in untreated maternal thyroid disorders, alterations in TH action within the placental compartment is postulated to contribute to the pathophysiology of adverse outcomes associated with malplacentation (14), such as miscarriages, preeclampsia, abruption, intrauterine growth restriction, postpartum hemorrhage, and stillbirths.

The human placenta is hemochorial, consisting of chorionic villi, which are in direct contact with maternal blood. Chorionic villi are entirely covered by a cellular layer of syncytiotrophoblast, which forms the primary placental cell barrier to maternal-fetal exchange (15) and necessitates the transcellular passage of almost all compounds, including THs (16). After passage through the syncytiotrophoblast, maternal THs have to traverse the cytotrophoblast layer (present as a continuous layer only in the first half of gestation), villous extracellular matrix, and capillary endothelium before entering the fetal circulation.

Transplacental TH passage is already known to be regulated by the catabolic activity of iodothyronine deiodinase type 3 in villous trophoblast (17), which plays a key role in protecting the fetus from excessive TH exposure (18). The activation of the predominant circulating TH, T_4_, into the active ligand, T_3_, by deiodinase type 2 in villous trophoblast is postulated to provide for local TH action. However, a role in supplying T_3_ to the fetus cannot be excluded (17). In addition, we hypothesize that the activity of plasma membrane TH transporters in trophoblast plays a critical part in the physiological regulation of maternal to fetal TH transfer (16). We have previously reported that the human placenta expresses a range of plasma membrane TH transporters, such as monocarboxylate transporter (MCT)8 [solute carrier gene family (SLC)16A2] and MCT10 [SLC16A10], organic anion-transporting polypeptide (OATP) 1A2 [SLCO1A2] and OATP4A1 [SLCO4A1], and the system-L amino acid transporters [LAT1 (SLC7A5), LAT2 (SLC7A8), and CD98 (SLC3A2)] from 6 wk of gestation until term, and these are localized in different placental cell types, including villous trophoblast (19, 20).

This study has focused on the “maternal-facing” apical microvillous plasma membrane (MVM) of human syncytiotrophoblast, which serves as the first plasma membrane barrier to the transplacental passage of maternal THs. We aimed to identify the range of TH transporters in MVM and to determine the contribution of each TH transporter to the uptake of the two major forms of TH, T_4_ and T_3_.

## Materials and Methods

### Human placental samples

Human placentae were obtained after written informed consent with ethical approval (South Birmingham Local Research Ethics Committee and the Research and Development Committee of the Birmingham Women's Hospital). Placentae (n = 22) were collected from uncomplicated, singleton pregnancies at term (38–41 wk; as determined by first trimester ultrasound scan) after delivery of normal birth weight infants (customized birth weight percentile 50.2 ± 6.1, mean ± sem) by elective cesarean section for previous cesarean section, breech presentation, or patient choice.

### Materials

All chemicals were purchased from either Sigma-Aldrich Co. Ltd. (Poole, UK) or VWR International (Lutterworth, UK) unless otherwise stated. L-[^125^I]-T_4_ (116 mCi/mmol^−1^) and L-[^125^I]L-T_3_ (97.3 mCi/mmol^−1^) were obtained from PerkinElmer (Beaconsfield, UK).

### MVM vesicle preparation

MVM vesicles were prepared from human placentae as described previously, using Mg^2+^ treatment to selectively aggregate nonmicrovillous plasma membranes, which can then be separated from MVM by centrifugation (21, 22). Briefly, villous placental tissue was homogenized, followed by two rounds of treatment with 10 mm MgCl_2_, and differential centrifugation. MVM vesicles were generated from the resultant final pellet, suspended in intravesicular buffer [290 mm sucrose, 5 mm Tris-base, and 5 mm HEPES (pH 7.4)] by application of a high shear force through a fine gauge needle to promote vesiculation and sealing of MVM plasma membrane fragments. Protein concentration was measured by adapted Bradford protein assay. MVM purity was assessed by determining the enrichment of alkaline phosphatase, which has a highly polarized distribution to MVM of the syncytiotrophoblast (23), as measured by its activity in MVM vesicles compared with the initial homogenate (21). Overall, alkaline phosphatase activity was enriched 29.9 ± 1.3-fold in MVM vesicles (mean ± sem, n = 22). Vesicles were suspended in intravesicular buffer and stored at 4 C before transport assay, which were performed within 72 h of isolation. Alternatively, some vesicles were stored in liquid nitrogen for further transport experiments later. Repeat uptake experiments after storage demonstrated that this did not affect MVM transport ability (data not shown). The correct orientation of the vesicles was confirmed on a subset of samples (n = 7) by measuring alkaline phosphatase-specific activity before and after permeabilization with 0.1% saponin (mean ± sem were 0.107 ± 0.015 and 0.104 ± 0.014 change of absorbance at 410 nm per 2 min/mg protein, respectively), revealing that MVM vesicles were the right-side-out, as shown previously (22).

### Western immunoblots

Western blot analysis was performed as we have described previously (19, 20). Proteins were extracted from four paired samples of placental homogenate and corresponding MVM vesicle isolates. Briefly, placental tissues were homogenized in lysis buffer [100 mmol/liter NaCl, 0.1% Triton X-100, and 50 mmol/liter Tris (pH 8.3)] containing protease inhibitors (1 mmol/liter phenylmethylsulfonylfluoride, 0.3 μmol/liter aprotinin, and 0.4 mmol/liter leupeptin). Protein samples (75 μg) were denatured (1 h, room temperature) in loading buffer (Laemmli sample buffer with dithiothreitol at 54 mg/ml), separated by electrophoresis in 10% SDS-PAGE gels, and blotted on nitrocellulose membranes. Blots were then probed with primary antibody: rabbit polyclonal antisera to human MCT8 (Ab4790) (9), MCT10 (Ab2198) (24), OATP1A2 or OATP4A1 (kind gift from Bruno Stieger, University Hospital of Zürich, Zürich, Switzerland) (25), LAT1 (26), or antihuman CD98 goat polyclonal antibody (Santa Cruz Biotechnology, Inc., Santa Cruz, CA). Primary antibodies were used at 1:500 dilutions, with the exception of the anti-CD98 at 1:250, with overnight incubation at 4 C. Blots were then probed with secondary antibody conjugated to horseradish peroxidase and bands visualized using the enhanced chemiluminescence detection kit (GE Healthcare Life Sciences, Princeton, NJ). Blots were reprobed for β-actin (1:10,000; Sigma, St. Louis, MO) to assess protein loading. Primary antibody specificities were as previously demonstrated in human placental tissue (19, 24). There was no cross-reactivity between MCT8 and MCT10 antibodies; each exhibited specificity to their target protein (Supplemental Fig. 1, published on The Endocrine Society's Journals Online web site at http://endo.endojournals.org).

### TH uptake assays

#### Kinetics of TH transport in MVM of human placenta

TH uptake assay methods were developed based upon those previously used to assess amino acid uptake (22). Uptake of 10 nm [^125^I]L-T_4_ and [^125^I]L-T_3_ was performed at room temperature in duplicate and initiated by combining 20 μl of MVM vesicle (10 mg protein/ml) in intravesicular buffer with 20 μl of extravesicular buffer (EVB) [5 mm HEPES, 5 mm Tris, and either 145 mm NaCl or KCl (pH 7.4)] containing the tracer. Uptake was stopped at different time points (15 sec to 60 min) by the addition of 2 ml of ice-cold stop solution [Krebs ringer phosphate buffer: 130 mm NaCl, 10 mm Na_2_HPO_4_, 4.2 mm KCl, 1.2 mm MgSO_4_, and 0.75 mm CaCl_2_ (pH 7.4)] and filtered through a HAWP nitrocellulose filter (0.45 mm; Millipore, Bedford, MA). The radioactivity on the filters was determined with a γ-counter (Wallac 1260 Multigamma II; Wallac, Turku, Finland). Binding (specific and nonspecific) of tracer to MVM plasma membrane was quantified after disruption of vesicle integrity with 0.25% Triton X-100. Results are expressed as picomoles of tracer per milligram of protein.

#### Effects of inhibitors of TH transporters

Radiolabeled [^125^I]L-T_4_ and [^125^I]L-T_3_ uptake studies were performed in the presence of unlabeled substrates and known inhibitors of the identified TH transporters: T_4_, T_3_, rT_3_, and triiodothyroacetic acid (TRIAC) at 10 μm; L-Trp, L-Phe, L-Leu, and 2-norbornanecarboxylic acid (BCH) at 10 mm; and bromosulfophthalein (BSP), probenecid (Prob), and desipramine (DMI) at 1 mm. We have used similar or higher concentrations of inhibitors compared with other TH uptake studies (26–34), which in turn are many fold higher than the respectively reported inhibitor dissociation constant *(K*_i_) values. No further inhibition of uptake was demonstrable using higher doses of inhibitors in preliminary dose-response experiments. The inhibitors T_4_, T_3_, rT_3_, and TRIAC were dissolved to 40 mm and Prob to 100 mm, respectively, in 0.1 n NaOH and BSP to 0.5 m in dimethylsulfoxide initially, then diluted further in EVB for uptake assays. All other inhibitors were dissolved directly in EVB. The BSP and Prob diluents did not show any effect on [^125^I]L-T_4_ and [^125^I]L-T_3_ uptake.

After the results of the initial kinetic experiments, [^125^I]L-T_4_ and [^125^I]L-T_3_ uptake was measured at 2 and 1 min, respectively, which correspond to the rapid linear phase of uptake. Within each experiment, inhibitors were tested in duplicate on each MVM vesicle isolate. Binding of the radiolabeled tracer to vesicle membrane itself was also measured in presence of 0.25% Triton X-100 and subtracted from the total amount of tracer per milligram or protein to quantify only the internalized tracer. Results are expressed as percentage of uptake compared with the no inhibitor control within each experiment. In addition, results of the effects of competitive inhibitors are also expressed in the text as a percentage of inhibition relative to that in the presence of excess cold T_4_ or T_3_, *i.e*. saturable uptake or T_4_-inhibitable [^125^I]L-T_4_ or T_3_-inhibitable [^125^I]L-T_3_ uptake.

### Statistical analysis

Data were analyzed using GraphPad Prism statistical software (version 4; GraphPad, San Diego, CA). The nonparametric one-way ANOVA, Kruskal-Wallis test, was used to analyze the effect of the various inhibitors on T_4_ and T_3_ uptake followed by Dunn's multiple comparison *post hoc* tests to compare each experimental group with the no inhibitor control.

## Results

### Expression of TH transporters in MVM vesicles

**Fig. 1.**
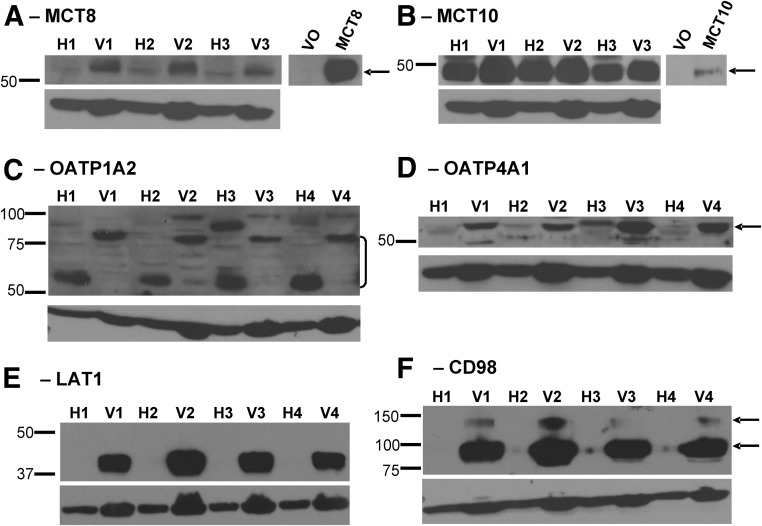
Western blot analysis for TH transporters in the MVM of human syncytiotrophoblasts. Protein was extracted from placental homogenate (H) and corresponding MVM vesicle isolates (V) from four representative placentae (1–4). Bands were observed at approximately 60 kDa for MCT8 (A), approximately 50 kDa for MCT10 (B), between 60 and 85 kDa representing different glycosylated states of OATP1A2 (C), at approximately 60 kDa for OATP4A1 (D), at 35 kDa for LAT1 (E), and two distinct species of CD98 at 85 and 135 kDa (F). Specificity of the MCT8 (A) and MCT10 (B) polyclonal antibody was tested, respectively, in JEG3 cells and HTR8 cells transfected with empty vector (VO), MCT8 plasmid (MCT8), or MCT10 plasmid (MCT10). β-Actin was used to assess protein loading and sample integrity. *Arrows* denote specific immunoreactive species.

We first confirmed the expression of a range of TH transporters in the MVM (Fig. 1). The vesicle preparations showed positive immunoreactivity for MCT8, MCT10, OATP1A2, OATP4A1, LAT1, and CD98 protein. This confirms previous immunohistochemistry findings in the human placenta, which had localized these transporters to the syncytiotrophoblast at term, with many studies describing a high concentration of expression at the apical membrane of syncytiotrophoblast (19, 20, 26, 35, 36). Specifically, the presence of system-L (LAT-CD98) has previously been inferred through the demonstration of BCH-inhibitable L-Ser uptake by MVM vesicles (37, 38).

Even though the same amount of protein was loaded into each well, the proportion of β-actin in MVM vesicle protein extracts is likely to differ from whole-cell protein extracts of placental homogenates. β-Actin can, thus, only confirm sample integrity and equal loading among MVM samples and among whole-cell homogenates, respectively. Thus, any apparent enrichment of protein in MVM fractions compared with homogenates is being interpreted with caution. Among the MVM samples, there was a readily detectable and consistent amount of protein expression of all the TH transporters being investigated. Differentially glycosylated OATP1A2 proteins of similar sizes have previously been detected with the same antibody in placental homogenates (19), brain (39), and liver (40). Interestingly, the more highly glycosylated OATP1A2 protein (∼85 kDa) appeared to be more concentrated in MVM vesicles compared with the less glycosylated forms (60–75 kDa), which were more heavily expressed in the placental homogenates. This suggests a diversity of locations and roles for OATP1A2 within the placenta associated with the degree of glycosylation. Two distinct species of CD98 at 85 and 135 kDa were detectable, which could represent different glycosylated states or incomplete disruption of disulfide bonds. Longer exposure of the blots probed for LAT1 and CD98 allowed the detection of LAT1 and CD98 proteins in placental homogenates (data not shown), suggesting significant enrichment of these proteins in the MVM fraction. By comparison with other transporters, apparent enrichment of MCT10 in MVM fractions was less marked, which may be due to its relative abundance in cytotrophoblasts (19) and the fetal-facing syncytiotrophoblast basal plasma membrane (41).

### Characterization of TH transport by microvillous plasma membrane vesicles

**Fig. 2.**
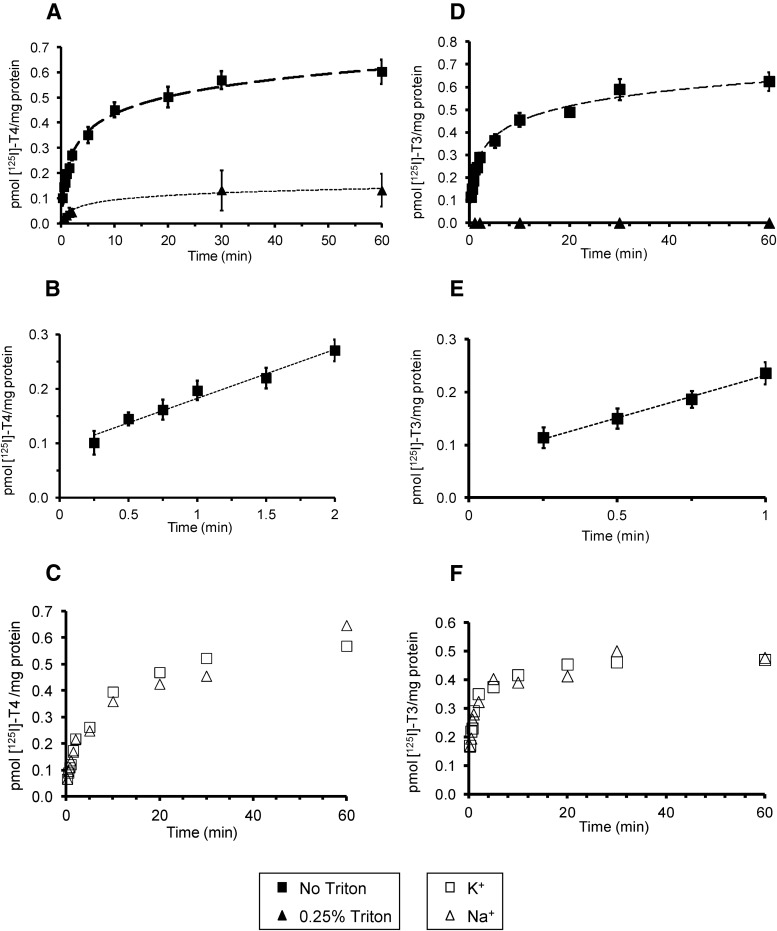
Characterization of T_4_ (A–C) and T_3_ (D–F) uptake by the MVM of human syncytiotrophoblasts. A and D, Time-dependent uptake of 10 nm [^125^I]L-T_4_ and [^125^I]L-T_3_ into MVM vesicles over 15 sec to 60 min (■). Data are mean ± sem (r^2^ > 0.9 for both). Disruption of vesicle integrity with 0.25% Triton X-100 significantly reduced [^125^I]L-T_4_ and [^125^I]L-T_3_ measurements (▴). B and E, Linearity of [^125^I]L-T_4_ and [^125^I]L-T_3_ uptake into MVM vesicles, respectively. Data are mean ± sem with linear regression plots shown (r^2^ > 0.9 for both). C and F, Lack of Na^+^ dependency of [^125^I]L-T_4_ and [^125^I]L-T_3_ uptake into MVM vesicles, assessed by measuring uptake in the presence (▵) and absence of Na (□). The *graphs* show one representative experiment.

We initially examined the basic kinetics of [^125^I]L-T_4_ and [^125^I]L-T_3_ uptake into MVM vesicles. Uptake of 10 nm [^125^I]L-T_4_ and [^125^I]L-T_3_ into MVM vesicles was time dependent (Fig. 2, A and D) and linear over the first 0.25–2 and 0.25–1 min, respectively (Fig. 2, B and E). Equilibrium was reached after 20 min of incubation for both [^125^I]L-T_4_ and [^125^I]L-T_3_ uptakes. The rates of [^125^I]L-T_4_ and [^125^I]L-T_3_ uptake during the linear phase were of similar magnitude at 0.09 pmol/mg protein per minute and 0.16 pmol/mg protein per minute, respectively. These are consistent with the previously reported uptake of T_3_ (50 nm) into BeWo choriocarcinoma cells (a model of villous trophoblasts), which is linear for 5 min at 37 C (26). However, T_4_ and T_3_ uptake by MVM is still slower than the MVM uptake of glucose (via GLUT glucose transporters) and amino acids (via systems L and A), where the linear phase occurs over approximately 1–3 sec at 23 C and 20–50 sec at 37 C, respectively (37, 42)

Experiments disrupting the vesicle integrity with 0.25% Triton X-100 indicated that there was significant binding of [^125^I]L-T_4_ to the MVM plasma membrane itself (Fig. 2A). [^125^I]L-T_4_ membrane binding could be due to specific and nonspecific binding to the MVM plasma membrane and/or binding of the tracer to transporters before TH translocation across the MVM plasma membrane. At the 2-min time point, [^125^I]L-T_4_ counts in the presence of Triton X-100 were 13% of that after [^125^I]L-T_4_ uptake without Triton X-100 (0.037 ± 0.009 and 0.291 ± 0.026 pmol/mg protein, respectively, n = 18). Contrary to [^125^I]L-T_4_, no binding of [^125^I]L-T_3_ to MVM plasma membrane was detected at any time point (1, 2, 10, 30, and 60 min) (Fig. 2D). Finally, [^125^I]L-T_4_ and [^125^I]L-T_3_ uptakes were both independent of Na^+^ as shown by the similar time course observed in the presence and absence of an inwardly directed Na^+^ gradient (Fig. 2, C and F). This suggests that sodium-dependent TH transporters, such as the Na^+^/taurocholate cotransporting polypeptide, are not involved in TH uptake by syncytiotrophoblast and indeed this transporter is absent from the placenta (43).

### Inhibition of radiolabeled TH uptake

To characterize the TH transporters mediating L-T_4_ and L-T_3_ uptake across the syncytiotrophoblast MVM, we measured [^125^I]L-T_4_ and [^125^I]L-T_3_ uptake into MVM vesicles in the presence of different TH metabolites (T_4_, T_3_, rT_3_ and TRIAC) and known competitive inhibitors of TH transport and substrates for MCT8, MCT10, system-L, and OATPs (Table 1). All the inhibitors tested could affect the activity of more than one of the identified TH transporter groups except BCH and Prob, which are known to inhibit only system-L and OATP activities, respectively. Furthermore, different competitive inhibitors of each transporter are not equally effective in their inhibition of TH uptake. For example, tryptophan (Trp) is more effective at inhibiting T_3_ uptake by MCT10 and system-L than phenylalanine (Phe) (29) and BCH (44), respectively. These two factors have to be taken into account in the interpretation of our results.

**Table 1.** Known TH transporter inhibitors and their targets

Inhibitor	MCT8	MCT10	System-L	OATPs
L-T_4_	✓	✓	✓	✓
L-T_3_	✓	✓	✓	✓
rT_3_	✓	✓	✓	✓
TRIAC	✓	✓	x	✓
L-Trp	x	✓	✓	x
L-Leu	x	x	✓	x
L-Phe	x	✓	✓	x
BCH	x	x	✓	x
BSP	✓	✓	x	✓
Prob	x	x	x	✓
DMI	✓	✓	✓	?

The inhibitors tested could affect the activity (✓) of more than one of the identified TH transporters except BCH and Prob, which are known to only inhibit system-L and OATP activity, respectively. The competitive inhibitors of each transporter are not equally effective in their inhibition of TH uptake, and this may also differ between different cells and tissue type (26–34, 48). Where inhibitors are not known to affect TH transport by the identified TH transporters is indicated by a *letter ex*. The effect of DMI on OATP activity is not known (*question mark*).

### Inhibition of [^125^I]L-T_4_ uptake

**Fig. 3.**
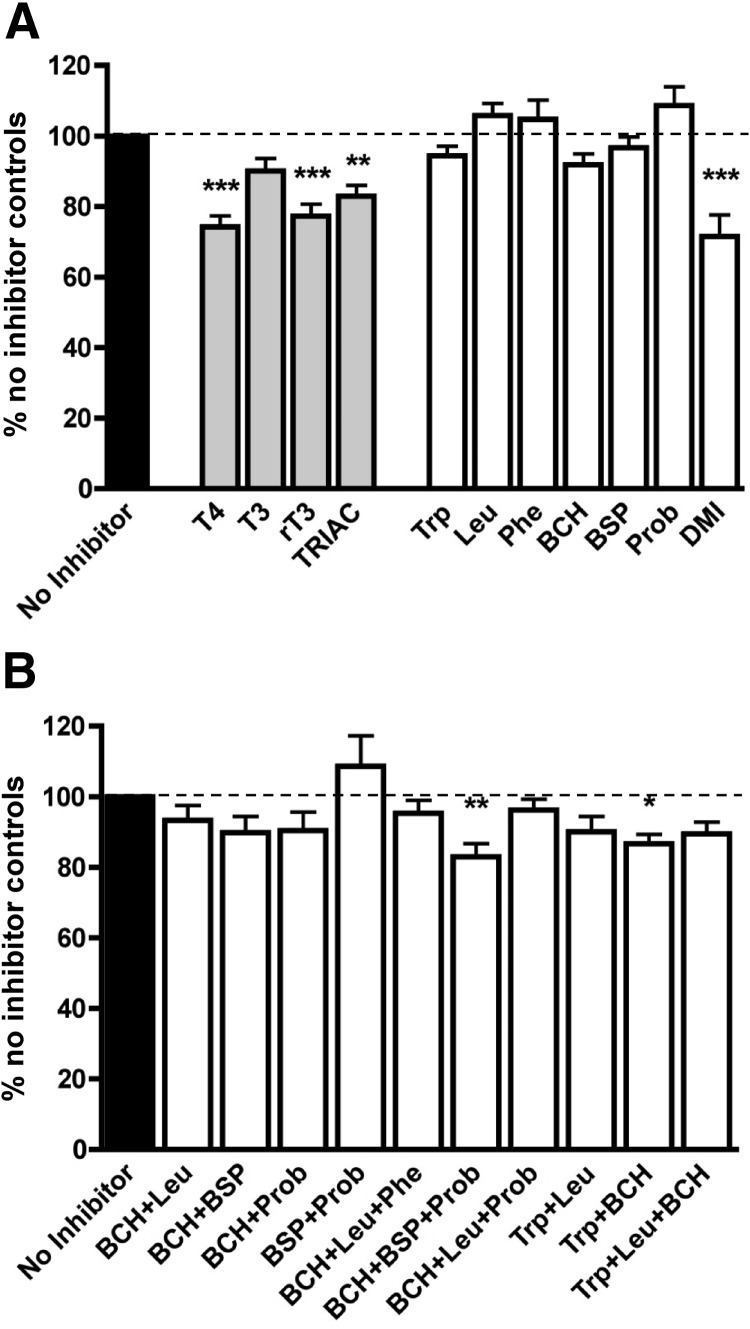
Inhibition of T_4_ uptake by known TH transporter inhibitors. [^125^I]L-T_4_ uptake assays were performed at 2 min in the presence of single inhibitors on their own (n = 8–9 for each inhibitor) (A) or in combination (n = 6 for each combination) (B). Data are mean + sem. Binding of [^125^I]L-T_4_ to MVM measured in presence of Triton X-100 within each experiment was subtracted. *, *P* < 0.05; **, *P* < 0.01; ***, *P* < 0.001 *vs.* no inhibitor control. Other significant differences occurred between leucine (Leu), Phe, and Prob with each of T_4_, rT_3_, TRIAC, and DMI in A (not indicated). Multiple comparisons between combinations of inhibitors in B showed no other significant differences.

Excess of cold T_4_ (10 μm) significantly reduced [^125^I]L-T_4_ uptake by 26% compared with no inhibitor controls (n = 8, *P* < 0.001) (Fig. 3A). A large proportion of [^125^I]L-T_4_ uptake could not be inhibited. Cold T_3_, however, did not significantly affect [^125^I]L-T_4_ uptake (n = 8, *P* = not significant). Compared with no inhibitor controls, rT_3_ and TRIAC reduced T_4_ uptake by 23% (n = 8, *P* < 0.001) and 17% (n = 8, *P* < 0.01), respectively.

None of the competitive inhibitors of TH transport tested had a significant effect on [^125^I]L-T_4_ uptake individually (Fig. 3A), suggesting that no single transporter plays a dominant role. Only DMI, a noncompetitive inhibitor of T_3_ transport mediated by MCT8, MCT10 (33), and an inhibitor of Trp transport mediated by system-L (44), could, on its own, significantly inhibit [^125^I]L-T_4_ uptake by 29% compared with no inhibitor controls (n = 9, *P* < 0.001), a similar degree to excess T_4_. DMI could be physically limiting the access of T_4_ to its transporters.

We then investigated the effects of combinations of competitive inhibitors on [^125^I]L-T_4_ uptake (Fig. 3B). Combination of the two competitive inhibitors, BCH (specific system-L inhibitor) and Trp (MCT10 and system-L inhibitor), which had individually shown the most reproducible and greatest inhibition (although not significant) of [^125^I]L-T_4_ uptake, exhibited an additive effect. Together, BCH and Trp significantly reduced [^125^I]L-T_4_ uptake in MVM by 14% compared with no inhibitor controls, which is equivalent to 54% inhibition of T_4_-inhibitable, or “saturable,” [^125^I]L-T_4_ uptake (n = 6, *P* < 0.05). Combining BCH with the broad spectrum competitive inhibitor, BSP (MCT10, MCT8, and OATP inhibitor), did not demonstrate a statistically significant effect. However, the addition of Prob (broad spectrum OATP inhibitor) to the combination of BCH and BSP resulted in a significant inhibition of 17% compared with no inhibitor controls (67% of saturable T_4_ uptake; n = 6, *P* < 0.01). Other combinations of competitive inhibitors did not show any significant effects. Significant inhibition of [^125^I]L-T_4_ uptake could only be achieved by combinations of inhibitors that could effectively inhibit at least system-L and MCT10 simultaneously. Taken together, these data indicate that saturable T_4_ uptake may be largely facilitated by system-L and MCT10 with a minor role played by OATPs.

### Inhibition of [^125^I]L-T_3_ uptake

**Fig. 4.**
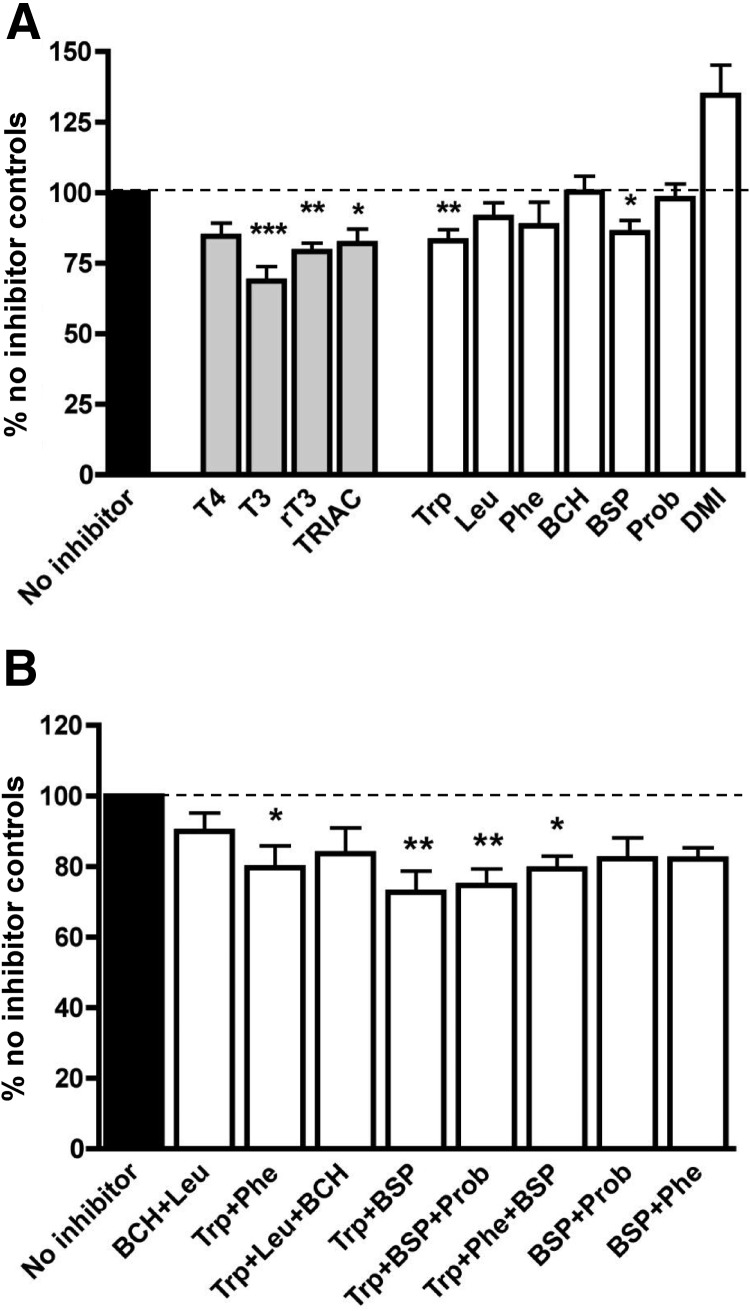
Inhibition of T_3_ uptake by known TH transporter inhibitors. [^125^I]L-T_3_ uptake assays were performed at 1 min in the presence of single inhibitors on their own (n = 7 for each inhibitor, n = 18 for DMI) (A) or in combination (n = 6 for each combination) (B). Data are mean + sem. Binding of [^125^I]L-T_3_ to MVM measured in presence of Triton X-100 within each experiment was subtracted. *, *P* < 0.05; **, *P* < 0.01; ***, *P* < 0.001 *vs.* no inhibitor control. Other significant differences occurred between DMI with each of T_4_, T_3_, rT_3_, TRIAC, Trp, and BSP in A (not indicated). Multiple comparisons between combinations of inhibitors in B showed no other significant differences.

As observed with T_4_ uptake, an excess of cold T_3_ (10 μm), could significantly reduce [^125^I]L-T_3_ uptake by 31% compared with no inhibitor controls (n = 8, *P* < 0.001) (Fig. 4A). Similarly, a large proportion of [^125^I]L-T_3_ uptake was not sensitive to inhibition. Compared with no inhibitor controls, rT_3_ and TRIAC reduced [^125^I]L-T_3_ uptake by 21% (n = 7, *P* < 0.01) and 18% (n = 7, *P* < 0.05), respectively, whereas cold T_4_ did not significantly affect [^125^I]L-T_3_ uptake (n = 7, *P* = not significant).

Among the competitive inhibitors of TH transport tested, Trp (MCT10 and system-L inhibitor) and BSP (MCT10, MCT8, and OATP inhibitor) could individually inhibit [^125^I]L-T_3_ uptake by 17% compared with no inhibitor controls (55% of saturable T_3_ uptake; n = 8, *P* < 0.01) and by 14% compared with no inhibitor controls (45% of saturable T_3_ uptake; n = 8, *P* < 0.05), respectively (Fig. 4A). The noncompetitive inhibitor, DMI, did not inhibit [^125^I]L-T_3_ uptake (n = 18, *P* = not significant compared with no inhibitor), which is in contrast to neurons (33). This may be associated with differences in the characteristics of the local membrane environment, including the profile of accessory membrane proteins and TH-binding proteins, which could affect DMI's access and binding to specific transporters but not others.

Combination of the two competitive inhibitors, Trp and BSP, which individually showed a significant effect on [^125^I]L-T_3_ uptake, exhibited an additive effect, greater than that compared with Trp or BSP alone. Together, they significantly reduced [^125^I]L-T_3_ uptake by 27% compared with no inhibitor controls (87% of saturable T_3_ uptake; n = 6, *P* < 0.01). Addition of Prob (broad spectrum OATP inhibitor) or Phe (MCT10 and system-L inhibitor) could not increase further the inhibitory effect of the Trp and BSP combination, inhibiting 25 and 21% of [^125^I]L-T_3_ uptake, respectively, compared with no inhibitor controls (81 and 66% of saturable T_3_ uptake, respectively; n = 6 and *P* < 0.01 for both). The combination of Trp and Phe (both MCT10 and system-L inhibitors) could also inhibit 20% of [^125^I]L-T_3_ uptake compared with no inhibitor controls (65% of saturable T_3_ uptake; n = 6, *P* < 0.05). The fact that BCH (specific system-L inhibitor) and Prob (a broad spectrum OATP inhibitor) individually had no effect, and when each was added to Trp and/or BSP they could not inhibit T_3_ uptake further, suggests that system-L and OATPs do not play a major role in T_3_ uptake. Taken together, our results suggest that saturable T_3_ uptake is facilitated mainly by MCT8 and MCT10 in syncytiotrophoblast MVM.

## Discussion

These data have provided the first direct evidence that the maternal-facing apical MVM of human placental syncytiotrophoblasts is capable of rapid, saturable T_4_ and T_3_ uptake and, therefore, could take up THs directly from the maternal circulation *in vivo*. Our findings are consistent with a highly novel postulate that these two major forms of TH are taken up by largely different groups of transporters at the syncytiotrophoblast MVM. We have demonstrated that multiple TH transporters are involved in placental T_4_ and T_3_ uptake with no single one of them playing a dominant role.

The relatively modest degree of inhibition of T_4_ and T_3_ uptake into MVM vesicles by excess substrate, despite correction for binding of the tracer to the disrupted membrane after Triton treatment, indicates that a substantial part of TH transport at the MVM is not saturable under our experimental conditions. In *Xenopus* oocytes transfected with system-L or MCT8, only 50–75% of inhibition of T_4_ and T_3_ uptake could be achieved by excess unlabeled THs (28, 44). Similar results were reported in JaR (31), BeWo (26), and HepG2 cells (32) and rat liver sinusoidal membrane vesicles (30). In all of these experimental systems, a substantial degree of nonsaturable transport could be accounted for by the rapid partitioning of the amphipathic T_4_ and T_3_ compounds into the lipid bilayer, which would exaggerate assessments of uptake performed over a short time span. Furthermore, such partitioning can also be abolished by Triton disruption in our experimental model and would not be corrected for in our methodology. The possibility of TH uptake by simple diffusion across the plasma membrane could be reasonably dismissed given the large body of evidence to the contrary (45). Similarly, the integrity of MVM vesicles in our experimental model has been well documented (22), and poor integrity would not be a significant contributory factor to nonsaturable transport. Furthermore, the mere presence of TH transporters in the MVM is likely not to be the only factor responsible for the physiological regulation of TH uptake. It is also conceivable that *in vivo* syncytiotrophoblast, unlike the empty MVM vesicles used in this study, could compartmentalize THs into endosomes and nuclei, as well as possess intracellular TH binding proteins and enzymes that metabolize THs, which could promote T_4_ and T_3_ uptake further by reducing the submembranous cytosolic concentrations of TH, thus providing a steeper gradient across the membrane. Also, any possible ATP-dependent TH uptake would be absent in our MVM model, including endocytosis, although no such active transport of TH has ever been conclusively demonstrated to date. There are suggestions that the expression of transthyretin at the apical surface of syncytiotrophoblast may be involved in facilitating the internalization of T_4_ (46, 47), perhaps through endocytic mechanisms. Alternatively, such TH binding proteins could increase the local membrane surface concentration of TH to promote entry through transporters. Therefore, in consideration of all of these factors, total saturable physiological uptake of T_4_ and T_3_ at the syncytiotrophoblast MVM *in vivo* is likely to be greater than that demonstrated by this MVM model.

Interestingly, T_4_ could not significantly inhibit the uptake of T_3_ and *vice versa*, strongly suggesting that these two major forms of TH are taken up by largely different transporters at the syncytiotrophoblast MVM, as previously observed in HepG2 human hepatoma cells (32). However, other forms of TH, rT_3_ (which is abundant in the fetal circulation) and TRIAC (TH analog), could inhibit the uptake of T_4_ and T_3_, as has previously been reported in JaR choriocarcinoma cells (31) and HepG2 human hepatoma cells (32). Because TRIAC, which does not inhibit many amino acid transporters, could inhibit 67 and 58% of saturable T_4_ and T_3_ uptake into MVM vesicles, respectively, it suggests that more than half of saturable T_4_ and T_3_ uptake is not mediated by amino acid transporters such as system-L.

System-L, MCT10, MCT8, and OATP1A2 are all known to have a greater affinity for T_3_ uptake than T_4_ with Michaelis constant *(K*_m_) values within a similar range between 0.8 and 8 μm (27–29, 48). T_4_ and T_3_ uptake by MVM would also be influenced by the presence of the other substrates transported by TH transporters. The *K*_m_ values for large neutral amino acid transport by system-L, aromatic amino acid transport by MCT10, and for various substrates transported by OATPs are all between 10- and 100-fold greater than for T_4_ and T_3_, as we have previously summarized (19). Thus, under physiological conditions, T_4_ and T_3_ could compete favorably with other substrates at the syncytiotrophoblast MVM. The relative levels of transporters expressed at the MVM would be a major factor in determining their individual contribution to T_4_ and T_3_ uptake and could be determined using mass spectroscopy, which is beyond the scope of this study. The conclusions drawn in this study relies upon previous knowledge of the characteristics of each transporter (Table 1) derived from other experimental models, such as *Xenopus laevis* oocytes, where individual transporters could be tested independently. However, it remains unclear what determines the predominantly divergent groups of transporters facilitating T_4_ and T_3_ uptake at the MVM.

The inability of single TH transporter inhibitors to inhibit TH uptake in systems possessing multiple TH transporters is not unique. In mouse primary cortical neurons where MCT8, system-L, and several OATPs are expressed, only BSP on its own showed a significant inhibitory effect on T_3_ uptake. The inhibitors BCH (specific system-L inhibitor) and Prob (OATP inhibitor) individually only showed a consistent but small inhibition, which was not significant. The contribution of system-L and Oatps to murine neuronal T_3_ transport was only appreciated when the combination of inhibitors BSP + BCH + Prob was found to have an additive and significant effect (34). TH uptake via multiple transporter systems appears to also be the case in the syncytiotrophoblast. Because combinations of inhibitors of the known TH transporters could only inhibit at most 67 and 87% of saturable T_4_ and T_3_ uptake, respectively, in MVM vesicles, we cannot exclude the possibility that there may be other, yet to be identified, TH transporters operating in the MVM. Of note, in mouse primary cortical neurons, the maximum reduction in T_3_ uptake that could be achieved through the use of combinations of inhibitors was still less than 50% compared with controls with no inhibitors despite the presence of normal intracellular components (34).

This study has provided a mechanistic framework, which could also be used to characterize TH transport across the syncytiotrophoblast plasma membranes during the early part of gestation. This would be of interest, because it is postulated that maternal to fetal TH transfer is most critical at this time. In addition to TH uptake from the maternal circulation at the syncytiotrophoblast MVM, TH efflux from syncytiotrophoblast at the basal plasma membrane will also be required to mediate the transfer of TH from the mother to the fetus and this also warrants investigation.

In conclusion, we have demonstrated that at term multiple TH transporters are present in the syncytiotrophoblast MVM, which could facilitate saturable T_4_ and T_3_ uptake directly from the maternal circulation *in vivo*. This locus is a point of regulation, in addition to TH metabolism by placental deiodinases, for the transplacental transfer of TH from the mother to the fetus.
